# Molecular Mechanisms Underlying Sensory-Motor Circuit Dysfunction in SMA

**DOI:** 10.3389/fnmol.2019.00059

**Published:** 2019-03-04

**Authors:** Hannah K. Shorrock, Thomas H. Gillingwater, Ewout J. N. Groen

**Affiliations:** ^1^Edinburgh Medical School: Biomedical Sciences, The University of Edinburgh, Edinburgh, United Kingdom; ^2^Euan MacDonald Centre for Motor Neurone Disease Research, The University of Edinburgh, Edinburgh, United Kingdom

**Keywords:** spinal muscular atrophy, SMN, sensory-motor circuit, proprioception, motor neuron, neurodegenaration

## Abstract

Activation of skeletal muscle in response to acetylcholine release from the neuromuscular junction triggered by motor neuron firing forms the basis of all mammalian locomotion. Intricate feedback and control mechanisms, both from within the central nervous system and from sensory organs in the periphery, provide essential inputs that regulate and finetune motor neuron activity. Interestingly, in motor neuron diseases, such as spinal muscular atrophy (SMA), pathological studies in patients have identified alterations in multiple parts of the sensory-motor system. This has stimulated significant research efforts across a range of different animal models of SMA in order to understand these defects and their contribution to disease pathogenesis. Several recent studies have demonstrated that defects in sensory components of the sensory-motor system contribute to dysfunction of motor neurons early in the pathogenic process. In this review, we provide an overview of these findings, with a specific focus on studies that have provided mechanistic insights into the molecular processes that underlie dysfunction of the sensory-motor system in SMA. These findings highlight the role that cell types other than motor neurons play in SMA pathogenesis, and reinforce the need for therapeutic interventions that target and rescue the wide array of defects that occur in SMA.

## Introduction

**FIGURE 1:**
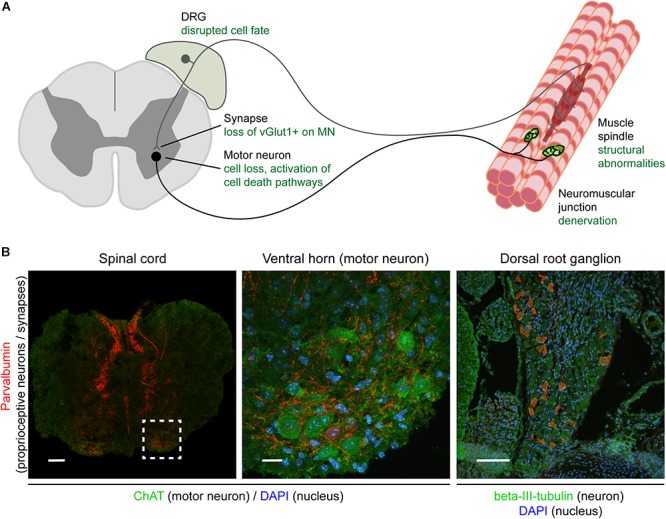
Overview of the sensory-motor system and pathologies observed in SMA. Schematic **(A)** and immunohistochemical **(B)** representation of the sensory-motor system, focusing on structures, cell types, and subcellular compartments that have been implicated in SMA pathogenesis. In **(A)**, the text in green indicates the primary pathological changes occurring in specific cell types or in specific subcellular compartments. In **(B)**, immunofluorescence was used to label parvalbumin-expressing cells and projections (proprioceptive neurons; red), neuronal cell bodies (ChAT for motor neurons, beta-III-tubulin for DRG cell bodies; green), and nuclei (DAPI; blue). Note that only a subgroup of DRG cell bodies express parvalbumin and that other markers such as NF200^+^ (mechano- and proprioceptive) and peripherin^+^ (nociceptive) can also be used to identify other types of sensory neurons. Scale bars: 100 μm (spinal cord and DRG); 20 μm (motor neuron). DRG, dorsal root ganglion; ChAT, acetylcholine transferase; DAPI, 4,6-diamidino-2-phenylindole; vGlut1, vesicular glutamate transporter 1; NF200, neurofilament heavy polypeptide (200 kDa).

Lower motor neurons, whose cell bodies are resident in the ventral gray horn of spinal cord, represent the “final common pathway” (Sherrington, 1906) through which neuronal activity has to pass in order to generate the contraction of skeletal muscle required for movement. Whilst a large proportion of the synaptic inputs controlling lower motor neuron function arise from descending pathways, including upper motor neurons, pioneering work begun by Sir John Eccles and colleagues in the 1950s has revealed the additional influence of synaptic inputs arising from proprioceptive sensory neurons (including group Ia afferents), whose cell bodies are located in the dorsal root ganglia (DRG) (Eccles et al., 1957; Brown, 1981; Mears and Frank, 1997). These sensory inputs have been shown to form monosynaptic connections with lower motor neurons (Figure 1), whereby they can regulate motor neuron firing patterns as part of the monosynaptic spinal stretch reflex arc as well as in other locomotor behaviors (Rossignol et al., 2006).

Recent work has begun to uncover the complex molecular pathways regulating the formation and maintenance of such sensory-motor connectivity in the spinal cord, highlighting key roles for pathways incorporating Sema3e–Plxnd1 signaling (Pecho-Vrieseling et al., 2009) and the RNaseIII protein Dicer (Imai et al., 2016). Moreover, several studies have highlighted how disruption of sensory-motor connectivity can result from perturbations in genes and proteins underlying a diverse range of neurodegenerative conditions, including; amyotrophic lateral sclerosis (ALS) (Jiang et al., 2009), post-polio muscular atrophy (PPMA) (Soliven and Maselli, 1992), and spinal muscular atrophy (SMA) (see below). In this review we provide an overview of the contributions that sensory-motor connectivity defects make to the pathogenesis of SMA, incorporating new insights into underlying molecular pathways that suggest the existence of common mechanisms across diverse neuromuscular conditions.

## Sensory-Motor Defects in Spinal Muscular Atrophy (SMA)

SMA is a hereditary form of motor neuron disease, characterized by degeneration and death of lower (alpha) motor neurons in the ventral horn of spinal cord (Lunn and Wang, 2008; Groen et al., 2018b). This leads to progressive proximal muscle weakness, atrophy and, in severe cases, paralysis and death. SMA is caused by homozygous deletion of, or other deleterious variants in, the *SMN1* gene, leading to a significant reduction in the expression of full-length survival motor neuron protein (SMN). SMN is a ubiquitously expressed protein that has been implicated in numerous cellular processes, including snRNP biogenesis, cytoskeletal dynamics, protein homeostasis, and mitochondrial function and is required for cellular survival. A detailed discussion of the cellular roles of SMN can be found in a number of recent review papers [for example (Hosseinibarkooie et al., 2017; Singh et al., 2017; Chaytow et al., 2018)].

In line with the ubiquitous expression of SMN, SMA is not solely a disease of the lower motor neuron. For example, it is now known that multiple different organ systems and cell types are affected in SMA (Nash et al., 2016), including defects in the heart, vasculature and skeletal muscles (Hamilton and Gillingwater, 2013; Shababi et al., 2014). Alongside these systemic pathologies, defects in sensory neurons have been reported in SMA patients, including abnormal sensory conduction (Duman et al., 2013; Yonekawa et al., 2013) or complete absence of sensory nerve action potentials (Yuan and Jiang, 2015; Reid et al., 2016), along with axonal degeneration and loss of myelinated fibers within sensory nerves (Korinthenberg et al., 1997; Omran et al., 1998; Rudnik-Schoneborn et al., 2003). Depending on the type of SMA, varying structural abnormalities in muscle spindles have been described, although some of these findings are contradictory and would require further investigation (Marshall and Duchen, 1975; Bobele et al., 1996; Kararizou et al., 2006). Several studies conducted on presumed SMA cases (patients diagnosed with SMA before genetic testing was available for the disease) also identified degeneration of sensory nerves, glial bundles within dorsal roots, ballooned neurons and chromatolysis within the DRG (Marshall and Duchen, 1975; Carpenter et al., 1978; Shishikura et al., 1983; Murayama et al., 1991). Importantly, reduced synaptophysin expression has been observed adjacent to anterior horn neuron cell bodies in SMA patients, indicating a reduction in the number of or disconnection of afferent nerve fibers, including sensory synapses (Ikemoto et al., 1996). Not only do these studies demonstrate defects within the sensory neurons themselves but they also demonstrate the possibility of alterations and lack of connectivity at both extremities of the sensory neuron.

### Sensory-Motor Defects in Mouse Models of SMA

The SMA research field has benefited greatly from the availability of a number of animal models that mimic key pathological features of SMA, such as motor neuron death, degeneration of the neuromuscular junction and organ pathology (Hamilton and Gillingwater, 2013; Edens et al., 2015). Indeed, model systems of SMA also recapitulate core defects of the sensory nervous system seen in SMA patients. For example, sensory neurons cultured from a severe mouse model of SMA show defects in neurite outgrowth and growth cone morphology, along with a reduction of beta-actin protein and mRNA in growth cones (Jablonka et al., 2006) – a phenotype that is similar to that observed in SMN deficient motor neurons (Rossoll et al., 2003). In mouse models of SMA, sensory neurons are smaller, consistent with an appearance of overall reduced size of DRGs (Shorrock et al., 2018b). Moreover, the ratio of different subtypes of sensory neurons in SMA DRG is altered (see Section “Molecular Mechanisms Associated With Sensory Neuron Dysfunction in SMA and Related Conditions”) (Shorrock et al., 2018b). It has also been shown that there is a reduction in myelinated dorsal root axons in SMA mice compared to controls (Ling et al., 2010) and a reduction of sensory fibers passing into the ventral horn in SMA mice (Mentis et al., 2011).

Importantly, in line with what has been observed in SMA patients, these structural defects occurring in sensory neurons have also been associated with defects at the level of synapses formed by sensory neurons in SMA mice (Fletcher and Mentis, 2016). The total number of synapses onto motor neurons in the lumbar region of the spinal cord is significantly reduced in mouse models of SMA (Ling et al., 2010). The largest contribution to this overall reduction of sensory synapses comes from a reduction in the number of vGlut1-positive synapses which primarily originate from proprioceptive neurons. This central feature of sensory-motor pathology has now been observed across multiple different SMA mouse models, including the commonly used *Taiwanese* and *delta7* models, suggesting that it is a conserved feature of the disease (Ling et al., 2010; Mentis et al., 2011; Shorrock et al., 2018b). Within the lumbar region of spinal cord, the reduction in proprioceptive synapses onto motor neurons is most severe at the level of L1 motor neurons and medial L5 motor neurons (Mentis et al., 2011). Interestingly, this reduction is observed presymptomatically, (Mentis et al., 2011; Fletcher et al., 2017) and is associated with significant functional deficits (Fletcher et al., 2017). Thus, sensory-motor connectivity defects reported in SMA mice consistently affect DRGs and motor neurons related to the body regions primarily affected in SMA patients, suggesting that defects occurring in SMA animal models are comparable to sensory-motor pathology observed in patients (Figure 1).

### SMN Expression Is Required Throughout the Sensory-Motor System

Whilst motor neuron death is central to the pathogenesis of SMA, in mouse models motor neuron death is preceded by dysfunction of motor neuron and neuromuscular transmission characterized by hyperexcitability, increased input resistance, and decreased synaptic efficacy (Ling et al., 2010; Mentis et al., 2011; Fletcher et al., 2017). These functional defects, in combination with pathological findings that show sensory neuron and synapse loss in SMA patients and mouse models, indicate a complex interplay between the various components of the sensory-motor system eventually leading to motor neuron defects and death.

As loss of SMN expression is central to SMA pathogenesis, recent research has aimed to determine the requirements for SMN expression in the sensory-motor system. In wild-type mice, SMN is expressed throughout all components of the sensory-motor system, including DRGs (Shorrock et al., 2018b), muscle, brain and spinal cord (Lee et al., 2012; Groen et al., 2018a), as well as peripheral nerves (Hunter et al., 2016). In *Drosophila* models of SMA, pan-neuronal restoration of SMN rescues locomotion and motor rhythm phenotypes (Imlach et al., 2012). In mouse models of SMA, pan-neuronal SMN restoration completely rescues motor neuron numbers and the number of vGlut1^+^ synapses onto motor neurons (Lee et al., 2012). To explore this phenomenon further, several research groups have investigated the requirements of SMN expression by restoring SMN in specific cell types of the sensory-motor system, using a range of genetic tools and animal lines (Gogliotti et al., 2012; Imlach et al., 2012; Martinez et al., 2012; Hao le et al., 2015; Simon et al., 2016; Fletcher et al., 2017). However, it remains difficult to generate a consensus from these studies, meaning that the exact cell-type and temporal requirements for SMN expression in the sensory-motor system remain to be fully determined (Table 1). Overall, however, these studies indicate that motor neuron *death* may be primarily due to cell-autonomous effects while the underlying cause of motor neuron *dysfunction* has a larger contribution from defects in proprioceptive neurons which provide a substantial non-cell autonomous component of motor neuron pathology. These findings highlight important issues when considering temporal and tissue specific requirements of SMN-targeted therapies for SMA as restoring SMN expression specifically in proprioceptive and motor neurons at the same time led to the biggest improvement in SMA-associated pathology compared to restoring SMN expression in either motor or proprioceptive neurons alone (Fletcher et al., 2017).

**Table 1:** Overview of studies investigating SMN expression requirements in various components of the sensory-motor system.

Model system	Neuron subtype	Driver(s)	Effect of neuron subtype specific SMN restoration on sensory-motor phenotypes	Reference
Mouse (severe)	Motor neuron	Hb9-Cre	Full rescue of vGlut1^+^ puncta per 100 μm perimeter of motor neuron soma	Gogliotti et al., 2012
			Complete rescue of cervical, thoracic and lumbar medial motor column motor neuron number	

Zebrafish	Motor neuron	mnx1/hb9 Cre	Partial rescue of dorsal root ganglion neuron number	Hao le et al., 2015
(maternal zygotic *smn* mutant)			Rescue of DRG neuron axon length	
			Complete rescue of motor axon branches total length	

Mouse (delta 7)	Motor neuron	Chat-Cre	Partial rescue of vGlut1^+^ synapses per motor neuron soma (L1)	Martinez et al., 2012
			Partial rescue of L1 motor neuron number	

Mouse (delta 7)	Motor neuron	Chat-Cre	No rescue of vGlut1^+^ synapses onto motor neurons (L2)	Fletcher et al., 2017
			No rescue of motor neuron firing frequency	
			Partial rescue of L2 motor neuron number	
	
	Proprioceptive neuron	Pv-Cre	Rescue of vGlut1^+^ synapses per motor neuron soma and dendrites (L2)	
			Correction of motor neuron firing frequency	
			No rescue of L2 motor neuron number	

Drosophila (*smn-/-*)	Motor neuron	OK371-Gal4; OK6-Gal4	No improvement of defective locomotion velocity, NMJ ePSPs or motor rhythm	Imlach et al., 2012
	
	Cholinergic neuron (including proprioceptive neurons)	Cha-Gal4	Complete rescue of defective locomotion velocity, NMJ ePSPs, and motor rhythm

Embryonic stem cell-derived	Motor neuron	*Smn* RNAi	Reduced motor neuron survival	Simon et al., 2016
motor circuit		*Smn* RNAi^∗^	No reduction of vGlut2 excitatory synapses onto motor neurons	
			No change in motor neuron hyperexcitability	
	
	Excitatory interneuron	*Smn* RNAi^#^	Loss of vGlut2 excitatory synapses onto motor neurons	
			Induced motor neuron hyperexcitability	


*ChAT, choline acetyltransferase; Cha, choline acetyltransferase; Pv, Parvalbumin alpha; Hb9/mnx1, Motor neuron and pancreas homeobox protein 1; Smn, survival motor neuron protein; RNAi, RNA interference; L1/2, lumbar segment 1/2; DRG, dorsal root ganglia; NMJ, neuromuscular junction; ePSPs, excitatory postsynaptic potentials. ^∗^co-cultured with wild-type excitatory interneurons; ^#^co-cultured with wild-type motor neurons*.

The importance of non-cell autonomous effects on motor neurons is further illustrated by several studies that have investigated the effect of defects intrinsic to specific types of sensory neurons on motor neuron function, in otherwise healthy animals. For example, blocking neurotransmission specifically in proprioceptive neurons in wild-type mice can cause severe motor defects, shortened lifespan and motor neuron dysfunction (Fletcher et al., 2017). Similarly, inhibiting cholinergic neuron activity in *Drosophila* causes reduced locomotion, increased spontaneous rhythmic motor activity and increased EPSPs at the neuromuscular junction by reducing excitatory cholinergic input to motor neurons (Imlach et al., 2012). Likewise, pharmacological inhibition of excitatory neurotransmission in an embryonic stem cell model of the motor circuit induced motor neuron hyperexcitability but not motor neuron death (Simon et al., 2016). Finally, mutations in the mechanosensitive ion channel responsible for mechanosensation of light touch and proprioception, *PIEZO2*, cause a neuromuscular disease characterized by muscle atrophy, aberrant muscle development and function, mild sensory involvement, delayed motor milestones, and scoliosis (Chesler et al., 2016; Delle Vedove et al., 2016). Together these studies indicate that defects within sensory neurons themselves can lead to both motor neuron and muscle dysfunction in the absence of defects intrinsic to motor neurons.

In summary, the above work illustrates that SMA mouse models reliably reflect pathological changes in the sensory-motor system of SMA patients. Moreover, it also illustrates the importance of homeostasis of the sensory-motor system: correct functioning of each of its parts is required for it to function correctly as a whole. Mechanistically, these studies have largely focused on motor neuron pathology and SMN requirements. Further molecular studies will be required to better understand the cellular pathways that are involved in regulating these pathological changes. In the next section of this review, we will focus on studies that have aimed to address this issue, also drawing on molecular insights obtained by studying other diseases of the sensory-motor system.

## Molecular Mechanisms Underlying Sensory-Motor Connectivity Defects in SMA

The presence of sensory-motor connectivity defects in SMA (as illustrated by the loss of proprioceptive vGlut1^+^ synapses), alterations in sensory neuron development, and the requirement for SMN expression in different neuronal subpopulations have all been clearly established (Figure 1). In SMA, SMN depletion is at the basis of each of these pathological changes. However, the molecular mechanisms that modulate these changes downstream of SMN depletion are still unclear. It is by further studying the changes downstream of SMN depletion that we will be able to better understand why certain cell types are more sensitive to SMN depletion than others, why defects in certain cellular pathways affect certain cell types more than others, and, ultimately, how SMN depletion leads to SMA, including disruption of sensory-motor connectivity.

### Molecular Mechanisms Linked to Motor Neuron Death and Pathology in SMA

As illustrated by the cell-type specific SMN requirements discussed above, pathways leading to motor neuron death appear to be largely cell autonomous. A better understanding of the molecular mechanisms associated with motor neuron death was gained from studies that compared gene expression profiles of vulnerable and resistant pools of motor neurons. These studies identified differences in basal bioenergetics profiles between vulnerable and resistant motor neuron pools (Boyd et al., 2017), as well as differences in activation of the cell death-regulating protein p53 and differential regulation of its downstream targets (Murray et al., 2015; Simon et al., 2017). Mechanistically, it has been shown that the alternative splicing of *Mdm2* and *Mdm4* downstream of and concurrently with disrupted snRNP biogenesis acts synergistically to induce p53 accumulation in SMA (Van Alstyne et al., 2018). However, despite rescuing the reduction of MN numbers seen in SMA mice, neither the inhibition of p53 signaling nor virus-mediated overexpression of full-length *Mdm2* and *Mdm4* is able to rescue vGlut1^+^-synapse loss (Simon et al., 2017; Van Alstyne et al., 2018).

Other factors have also been linked to motor circuit function in SMA, including stasimon (Lotti et al., 2012). Decreasing stasimon expression in zebrafish phenocopied motor axon branching defects seen in *smn* morpholino zebrafish, which in turn could be rescued by overexpressing stasimon. Similarly, in a *Drosophila* model of SMA, expressing stasimon in cholinergic (proprioceptive) neurons, but not motor neurons, rescued neurotransmitter release at the NMJ and muscle size defects (Lotti et al., 2012). All of these studies indicate that, while motor neuron death itself can be rescued by targeting the pathways that directly lead to motor neuron death (such as p53 activation, and Mdm2 and Mdm4 missplicing), this does not rescue motor neuron dysfunction or other motor neuron-associated phenotypes in models of SMA (Lotti et al., 2012; Van Alstyne et al., 2018). Other molecular factors such as agrin and plastin3 that are linked to and important for NMJ organization and AMPA receptor functioning have also been implicated in SMA (Zhang et al., 2013; Hosseinibarkooie et al., 2016; Kim et al., 2017). These factors could be important for the reduced synaptic transmission seen in SMA. Moreover, complement C1q, an important factor in postsynaptic pruning, is upregulated in SMA motor neurons (Zhang et al., 2013); a event which, along with an increased association of microglia with SMA motor neurons (Ling et al., 2010), might contribute to the impaired sensory-motor connectivity observed in SMA.

In summary, the above studies have provided important insights into the molecular mechanisms that underlie motor neuron death in SMA. However, in line with previous pathological and SMN expression studies (see Section “SMN Expression Is Required Throughout the Sensory-Motor System”), these studies also illustrate the importance of non cell-autonomous processes in sensory-motor dysfunction in SMA.

### Molecular Mechanisms Associated With Sensory Neuron Dysfunction in SMA and Related Conditions

The number of studies investigating the molecular mechanisms that underlie sensory neuron dysfunction in SMA is limited. Therefore, adapting biological findings generated from studies of other, related neuromuscular diseases that are characterized by sensory neuron dysfunction has been of benefit to the SMA research field. For example, Charcot-Marie-Tooth (CMT) disease is a hereditary motor and sensory neuropathy that, depending on its genetic cause, has a variable clinical presentation. CMT type 2D, caused by mutations in the tRNA synthetase *GARS* (glycine tRNA-ligase or GlyRS), has a predominant motor phenotype, although sensory defects are also present (Motley et al., 2010). In CMT2D, sensory defects have been shown to be linked to a disruption in sensory neuron fate, downstream of increased expression of mutant GARS (Sleigh et al., 2017). Specifically, when determining sensory neuron subtypes in DRGs from two CMT2D mouse models, fewer large cell body NF200-positive (neurofilament heavy) cell bodies were present in favor of an increased number of peripherin-positive, smaller neurons (Sleigh et al., 2017). Taking this observation into the SMA context provides a possible explanation for the loss of sensory synapses onto lower motor neurons that occurs in the SMA spinal cord, as a shift in sensory neuron cell fate would also lead to changes at the level of their synaptic contacts. Indeed, when investigating sensory neuron development in the *Taiwanese* mouse model of SMA, it was shown that a similar change in sensory neuron fate occurs when SMN expression levels are reduced: a decrease in the number of NF200-positive mechano- and proprioceptive sensory neurons is accompanied by an increase in the number of peripherin-positive nociceptive sensory neurons (Shorrock et al., 2018b). Correspondingly, protein levels of GARS were also found to be changed in the spinal cord of SMA mice, as well as in DRGs, in a way that is comparable to changes in GARS observed in mouse models of CMT2D.

The upregulation of GARS in SMA was shown to be regulated by the E1 ubiquitin-activating enzyme, UBA1. UBA1 expression has previously been shown to be significantly decreased in SMA from very early in the pathogenic process and is therapeutically targetable (Wishart et al., 2014; Powis et al., 2016). When investigating protein changes that occur downstream of UBA1, GARS expression was found to depend on UBA1 expression (Shorrock et al., 2018b). In line with this, GARS-dependent pathological features of SMA, including altered sensory neuron fate and loss of proprioceptive synapses in the spinal cord, were rescued when UBA1 levels were elevated using a gene therapy approach (Shorrock et al., 2018b). These findings provide an interesting pathological link between SMA and CMT2D, but also indicate possible molecular pathways that may underlie sensory-motor pathology in SMA and provide starting points for further research. For example, possible mechanistic links related to disruption of GARS-mediated pathways have been shown to involve dysfunctional Nrp1 / VEGF and Trk signaling (He et al., 2015; Sleigh et al., 2017). Mutant GARS can aberrantly bind these proteins and this aberrant binding is thought to disrupt their function. However, to what extent these pathways are relevant for sensory defects associated with SMA remains to be determined.

In addition to defects in Trk and Nrp1/VEGF signaling, mutations in GARS are known to lead to increased levels of alpha-tubulin acetylation (d’Ydewalle et al., 2011). Alpha-tubulin acetylation depends on HDAC activity (Zhang et al., 2003), and, interestingly, both a broad spectrum HDAC inhibitor (trichostatin A) as well as a specific HDAC6 inhibitor (tubastatin A) reversed increases in alpha-tubulin acetylation in a range of cellular and animal models of CMT2D (d’Ydewalle et al., 2011; Benoy et al., 2018; Mo et al., 2018). Intriguingly, the broad spectrum HDAC inhibitor trichostatin A was previously found to rescue vGlut1^+^-synapse loss on spinal motor neurons in the *delta7* model of SMA (Mentis et al., 2011). To what extent this process is also related to alpha-tubulin acetylation or is due to other effects of HDAC inhibition in SMA remains to be determined. However, this provides an intriguing possible link between HDAC inhibition, GARS function, DRG pathology and sensory synapse loss.

In summary, these findings illustrate how molecular pathways that were previously identified in other disorders, such as CMT2D, can assist discovery of novel mechanisms and therapeutic targets that underlie sensory-motor connectivity defects in SMA.

## Concluding Remarks

Motor neurons rely on a range of synaptic inputs, both from within the central nervous system and from sensory neurons projecting from the periphery, to regulate and finetune their activity. In SMA, multiple components of this sensory-motor system have been shown to be disrupted, contributing to motor neuron dysfunction and death. Several molecular pathways have now been identified that regulate sensory neuron dysfunction and these pathways form interesting novel targets for further study and potential therapy development.

The SMA research field is currently at a defining moment. The first disease-modifying therapy for SMA was recently approved, while other therapies are currently in advanced stages of clinical development (Shorrock et al., 2018a). However, the fact that current therapies are targeted to the nervous system specifically and do not benefit all patients equally, illustrates that fundamental SMA research is still needed, perhaps more than ever (Groen et al., 2018b). Furthering our understanding of the molecular mechanisms that underlie pathological changes occurring in specific cell types, such as sensory neurons, will aid the discovery of novel therapeutic strategies. Moreover, this process may be sped up by scrutinizing the existing knowledge from other, related disorders, as is illustrated by the recent identification of shared molecular mechanisms between SMA and CMT2D. Combining advances in these areas of research will be necessary to be able to eventually provide therapies that will benefit and support all SMA patients equally and efficiently.

## Author Contributions

HS and EG performed the literature search and generated the table and figure. HS, TG, and EG prepared and wrote the manuscript.

## Conflict of Interest Statement

The authors declare that the research was conducted in the absence of any commercial or financial relationships that could be construed as a potential conflict of interest.

## References

[B1] Benoy V. Van Helleputte L. Prior R. d’Ydewalle C. Haeck W. Geens N. (2018). HDAC6 is a therapeutic target in mutant GARS-induced Charcot-Marie-Tooth disease. *Brain* 141 673–687. 10.1093/brain/awx375 29415205 PMC5837793

[B2] Bobele G. B. Feeback D. L. Leech R. W. Brumback R. A. (1996). Hypertrophic intrafusal muscle fibers in infantile spinal muscular atrophy. *J. Child. Neurol.* 11 246–248. 10.1177/088307389601100318 8734032

[B3] Boyd P. J. Tu W. Y. Shorrock H. K. Groen E. J. N. Carter R. N. Powis R. A. (2017). Bioenergetic status modulates motor neuron vulnerability and pathogenesis in a zebrafish model of spinal muscular atrophy. *PLoS Genet.* 13:e1006744. 10.1371/journal.pgen.1006744 28426667 PMC5417717

[B4] Brown A. G. (1981). *Organization in the Spinal Cord : The Anatomy and Physiology of Identified Neurones.* New York, NY: Springer-Verlag 10.1007/978-1-4471-1305-8

[B5] Carpenter S. Karpati G. Rothman S. Watters G. Andermann F. (1978). Pathological involvement of primary sensory neurons in Werdnig-Hoffmann disease. *Acta Neuropathol.* 42 91–97. 10.1007/BF00690973 654890

[B6] Chaytow H. Huang Y. T. Gillingwater T. H. Faller K. M. E. (2018). The role of survival motor neuron protein (SMN) in protein homeostasis. *Cell Mol. Life Sci.* 75 3877–3894. 10.1007/s00018-018-2849-1 29872871 PMC6182345

[B7] Chesler A. T. Szczot M. Bharucha-Goebel D. Ceko M. Donkervoort S. Laubacher C. (2016). The Role of PIEZO2 in Human Mechanosensation. *N. Engl. J. Med.* 375 1355–1364. 10.1056/NEJMoa1602812 27653382 PMC5911918

[B8] Delle Vedove A. Storbeck M. Heller R. Holker I. Hebbar M. Shukla A. (2016). Biallelic loss of proprioception-related PIEZO2 causes muscular atrophy with perinatal respiratory distress, arthrogryposis, and scoliosis. *Am. J. Hum. Genet.* 99 1206–1216. 10.1016/j.ajhg.2016.09.019 27843126 PMC5097934

[B9] Duman O. Uysal H. Skjei K. L. Kizilay F. Karauzum S. Haspolat S. (2013). Sensorimotor polyneuropathy in patients with SMA type-1: electroneuromyographic findings. *Muscle Nerve* 48 117–121. 10.1002/mus.23722 23629817

[B10] d’Ydewalle C. Krishnan J. Chiheb D. M. Van Damme P. Irobi J. Kozikowski A. P. (2011). HDAC6 inhibitors reverse axonal loss in a mouse model of mutant HSPB1-induced charcot-marie-tooth disease. *Nat. Med.* 17 968–974. 10.1038/nm.2396 21785432

[B11] Eccles J. C. Eccles R. M. Lundberg A. (1957). The convergence of monosynaptic excitatory afferents on to many different species of alpha motoneurones. *J. Physiol.* 137 22–50. 10.1113/jphysiol.1957.sp005794 13439582 PMC1362996

[B12] Edens B. M. Ajroud-Driss S. Ma L. Ma Y. C. (2015). Molecular mechanisms and animal models of spinal muscular atrophy. *Biochim. Biophys. Acta* 1852 685–692. 10.1016/j.bbadis.2014.07.024 25088406 PMC12184990

[B13] Fletcher E. V. Mentis G. Z. (2016). “Motor circuit dysfunction in spinal muscular atrophy,” in *Spinal Muscular Atrophy* eds Sumner C. J. Paushkin S. Ko C. P. (London: Elsevier) 153–165.

[B14] Fletcher E. V. Simon C. M. Pagiazitis J. G. Chalif J. I. Vukojicic A. Drobac E. (2017). Reduced sensory synaptic excitation impairs motor neuron function via Kv2.1 in spinal muscular atrophy. *Nat. Neurosci.* 20 905–916. 10.1038/nn.4561 28504671 PMC5487291

[B15] Gogliotti R. G. Quinlan K. A. Barlow C. B. Heier C. R. Heckman C. J. Didonato C. J. (2012). Motor neuron rescue in spinal muscular atrophy mice demonstrates that sensory-motor defects are a consequence, not a cause, of motor neuron dysfunction. *J. Neurosci.* 32 3818–3829. 10.1523/JNEUROSCI.5775-11.2012 22423102 PMC3679185

[B16] Groen E. J. N. Perenthaler E. Courtney N. L. Jordan C. Y. Shorrock H. K. van der Hoorn D. (2018a). Temporal and tissue-specific variability of SMN protein levels in mouse models of spinal muscular atrophy. *Hum. Mol. Genet.* 27 2851–2862. 10.1093/hmg/ddy195 29790918 PMC6077828

[B17] Groen E. J. N. Talbot K. Gillingwater T. H. (2018b). Advances in therapy for spinal muscular atrophy: promises and challenges. *Nat. Rev. Neurol.* 14 214–224. 10.1038/nrneurol.2018.4 29422644

[B18] Hamilton G. Gillingwater T. H. (2013). Spinal muscular atrophy: going beyond the motor neuron. *Trends Mol. Med.* 19 40–50. 10.1016/j.molmed.2012.11.002 23228902

[B19] Hao le T. Duy P. Q. Jontes J. D. Beattie C. E. (2015). Motoneuron development influences dorsal root ganglia survival and Schwann cell development in a vertebrate model of spinal muscular atrophy. *Hum. Mol. Genet.* 24 346–360. 10.1093/hmg/ddu447 25180019 PMC4275068

[B20] He W. Bai G. Zhou H. Wei N. White N. M. Lauer J. (2015). CMT2D neuropathy is linked to the neomorphic binding activity of glycyl-tRNA synthetase. *Nature* 526 710–714. 10.1038/nature15510 26503042 PMC4754353

[B21] Hosseinibarkooie S. Peters M. Torres-Benito L. Rastetter R. H. Hupperich K. Hoffmann A. (2016). The power of human protective modifiers: PLS3 and CORO1C unravel impaired endocytosis in spinal muscular atrophy and rescue sma phenotype. *Am. J. Hum. Genet.* 99 647–665. 10.1016/j.ajhg.2016.07.014 27499521 PMC5011078

[B22] Hosseinibarkooie S. Schneider S. Wirth B. (2017). Advances in understanding the role of disease-associated proteins in spinal muscular atrophy. *Expert Rev. Proteomics* 14 581–592. 10.1080/14789450.2017.1345631 28635376

[B23] Hunter G. Powis R. A. Jones R. A. Groen E. J. Shorrock H. K. Lane F. M. (2016). Restoration of SMN in Schwann cells reverses myelination defects and improves neuromuscular function in spinal muscular atrophy. *Hum. Mol. Genet.* 25 2853–2861. 10.1093/hmg/ddw141 27170316 PMC5181642

[B24] Ikemoto A. Hirano A. Matsumoto S. Akiguchi I. Kimura J. (1996). Synaptophysin expression in the anterior horn of werdnig-hoffmann disease. *J. Neurol. Sci.* 136 94–100. 10.1016/0022-510X(95)00297-F 8815186

[B25] Imai F. Chen X. Weirauch M. T. Yoshida Y. (2016). Requirement for dicer in maintenance of monosynaptic sensory-motor circuits in the spinal cord. *Cell Rep.* 17 2163–2172. 10.1016/j.celrep.2016.10.083 27880894 PMC5152923

[B26] Imlach W. L. Beck E. S. Choi B. J. Lotti F. Pellizzoni L. McCabe B. D. (2012). SMN is required for sensory-motor circuit function in *Drosophila*. *Cell* 151 427–439. 10.1016/j.cell.2012.09.011 23063130 PMC3475188

[B27] Jablonka S. Karle K. Sandner B. Andreassi C. von Au K. Sendtner M. (2006). Distinct and overlapping alterations in motor and sensory neurons in a mouse model of spinal muscular atrophy. *Hum. Mol. Genet.* 15 511–518. 10.1093/hmg/ddi467 16396995

[B28] Jiang M. Schuster J. E. Fu R. Siddique T. Heckman C. J. (2009). Progressive changes in synaptic inputs to motoneurons in adult sacral spinal cord of a mouse model of amyotrophic lateral sclerosis. *J. Neurosci.* 29 15031–15038. 10.1523/JNEUROSCI.0574-09.2009 19955354 PMC3000669

[B29] Kararizou E. Manta P. Kalfakis N. Gkiatas K. Vassilopoulos D. (2006). Morphological and morphometrical study of human muscle spindles in Werdnig-Hoffmann disease (infantile spinal muscular atrophy type I). *Acta Histochem.* 108 265–269. 10.1016/j.acthis.2006.03.020 16730053

[B30] Kim J. K. Caine C. Awano T. Herbst R. Monani U. R. (2017). Motor neuronal repletion of the NMJ organizer, agrin, modulates the severity of the spinal muscular atrophy disease phenotype in model mice. *Hum. Mol. Genet.* 26 2377–2385. 10.1093/hmg/ddx124 28379354 PMC6074815

[B31] Korinthenberg R. Sauer M. Ketelsen U. P. Hanemann C. O. Stoll G. Graf M. (1997). Congenital axonal neuropathy caused by deletions in the spinal muscular atrophy region. *Ann. Neurol.* 42 364–368. 10.1002/ana.410420314 9307259

[B32] Lee A. J. Awano T. Park G. H. Monani U. R. (2012). Limited phenotypic effects of selectively augmenting the SMN protein in the neurons of a mouse model of severe spinal muscular atrophy. *PLoS One* 7:e46353. 10.1371/journal.pone.0046353 23029491 PMC3459898

[B33] Ling K. K. Lin M. Y. Zingg B. Feng Z. Ko C. P. (2010). Synaptic defects in the spinal and neuromuscular circuitry in a mouse model of spinal muscular atrophy. *PLoS One* 5:e15457. 10.1371/journal.pone.0015457 21085654 PMC2978709

[B34] Lotti F. Imlach W. L. Saieva L. Beck E. S. Hao le T. Li D. K. (2012). An SMN-dependent U12 splicing event essential for motor circuit function. *Cell* 151 440–454. 10.1016/j.cell.2012.09.012 23063131 PMC3474596

[B35] Lunn M. R. Wang C. H. (2008). Spinal muscular atrophy. *Lancet* 371 2120–2133. 10.1016/S0140-6736(08)60921-6 18572081

[B36] Marshall A. Duchen L. W. (1975). Sensory system involvement in infantile spinal muscular atrophy. *J. Neurol. Sci.* 26 349–359. 10.1016/0022-510X(75)90207-5 127019

[B37] Martinez T. L. Kong L. Wang X. Osborne M. A. Crowder M. E. Van Meerbeke J. P. (2012). Survival motor neuron protein in motor neurons determines synaptic integrity in spinal muscular atrophy. *J. Neurosci.* 32 8703–8715. 10.1523/JNEUROSCI.0204-12.2012 22723710 PMC3462658

[B38] Mears S. C. Frank E. (1997). Formation of specific monosynaptic connections between muscle spindle afferents and motoneurons in the mouse. *J. Neurosci.* 17 3128–3135. 10.1523/JNEUROSCI.17-09-03128.1997 9096147 PMC6573627

[B39] Mentis G. Z. Blivis D. Liu W. Drobac E. Crowder M. E. Kong L. (2011). Early functional impairment of sensory-motor connectivity in a mouse model of spinal muscular atrophy. *Neuron* 69 453–467. 10.1016/j.neuron.2010.12.032 21315257 PMC3044334

[B40] Mo Z. Zhao X. Liu H. Hu Q. Chen X. Q. Pham J. (2018). Aberrant GlyRS-HDAC6 interaction linked to axonal transport deficits in Charcot-Marie-Tooth neuropathy. *Nat. Commun.* 9:1007. 10.1038/s41467-018-03461-z 29520015 PMC5843656

[B41] Motley W. W. Talbot K. Fischbeck K. H. (2010). GARS axonopathy: not every neuron’s cup of tRNA. *Trends Neurosci.* 33 59–66. 10.1016/j.tins.2009.11.001 20152552 PMC2822721

[B42] Murayama S. Bouldin T. W. Suzuki K. (1991). Immunocytochemical and ultrastructural studies of werdnig-hoffmann disease. *Acta Neuropathol.* 81 408–417. 10.1007/BF00293462 1851364

[B43] Murray L. M. Beauvais A. Gibeault S. Courtney N. L. Kothary R. (2015). Transcriptional profiling of differentially vulnerable motor neurons at pre-symptomatic stage in the Smn (2b/-) mouse model of spinal muscular atrophy. *Acta Neuropathol. Commun.* 3:55. 10.1186/s40478-015-0231-1 26374403 PMC4570693

[B44] Nash L. A. Burns J. K. Chardon J. W. Kothary R. Parks R. J. (2016). Spinal muscular atrophy: more than a disease of motor neurons? *Curr. Mol. Med.* 16 779–792. 10.2174/1566524016666161128113338 27894243

[B45] Omran H. Ketelsen U. P. Heinen F. Sauer M. Rudnik-Schoneborn S. Wirth B. (1998). Axonal neuropathy and predominance of type II myofibers in infantile spinal muscular atrophy. *J. Child. Neurol.* 13 327–331. 10.1177/088307389801300704 9701481

[B46] Pecho-Vrieseling E. Sigrist M. Yoshida Y. Jessell T. M. Arber S. (2009). Specificity of sensory-motor connections encoded by Sema3e-Plxnd1 recognition. *Nature* 459 842–846. 10.1038/nature08000 19421194 PMC2847258

[B47] Powis R. A. Karyka E. Boyd P. Come J. Jones R. A. Zheng Y. (2016). Systemic restoration of UBA1 ameliorates disease in spinal muscular atrophy. *JCI Insight* 1:e87908. 10.1172/jci.insight.87908 27699224 PMC5033939

[B48] Reid D. Zinger Y. Raheja D. (2016). Sensory neuronopathy in spinal muscular atrophy: a case presentation. *J. Clin. Neuromuscul. Dis.* 18 44–46. 10.1097/CND.0000000000000124 27552391

[B49] Rossignol S. Dubuc R. Gossard J. P. (2006). Dynamic sensorimotor interactions in locomotion. *Physiol. Rev.* 86 89–154. 10.1152/physrev.00028.2005 16371596

[B50] Rossoll W. Jablonka S. Andreassi C. Kroning A. K. Karle K. Monani U. R. (2003). Smn, the spinal muscular atrophy-determining gene product, modulates axon growth and localization of beta-actin mRNA in growth cones of motoneurons. *J. Cell Biol.* 163 801–812. 10.1083/jcb.200304128 14623865 PMC2173668

[B51] Rudnik-Schoneborn S. Goebel H. H. Schlote W. Molaian S. Omran H. Ketelsen U. (2003). Classical infantile spinal muscular atrophy with SMN deficiency causes sensory neuronopathy. *Neurology* 60 983–987. 10.1212/01.WNL.0000052788.39340.45 12654964

[B52] Shababi M. Lorson C. L. Rudnik-Schoneborn S. S. (2014). Spinal muscular atrophy: a motor neuron disorder or a multi-organ disease? *J. Anat.* 224 15–28. 10.1111/joa.12083 23876144 PMC3867883

[B53] Sherrington C. S. (1906). *The Integrative Action of the Nervous System.* New York, NY: C. Scribner’s sons.

[B54] Shishikura K. Hara M. Sasaki Y. Misugi K. (1983). A neuropathologic study of Werdnig-Hoffmann disease with special reference to the thalamus and posterior roots. *Acta Neuropathol.* 60 99–106. 10.1007/BF00685353 6880628

[B55] Shorrock H. K. Gillingwater T. H. Groen E. J. N. (2018a). Overview of current drugs and molecules in development for spinal muscular atrophy therapy. *Drugs* 78 293–305. 10.1007/s40265-018-0868-8 29380287 PMC5829132

[B56] Shorrock H. K. van der Hoorn D. Boyd P. J. Llavero Hurtado M. Lamont D. J. Wirth B. (2018b). UBA1/GARS-dependent pathways drive sensory-motor connectivity defects in spinal muscular atrophy. *Brain* 141 2878–2894. 10.1093/brain/awy237 30239612 PMC6158753

[B57] Simon C. M. Dai Y. Van Alstyne M. Koutsioumpa C. Pagiazitis J. G. Chalif J. I. (2017). Converging mechanisms of p53 activation drive motor neuron degeneration in spinal muscular atrophy. *Cell Rep.* 21 3767–3780. 10.1016/j.celrep.2017.12.003 29281826 PMC5747328

[B58] Simon C. M. Janas A. M. Lotti F. Tapia J. C. Pellizzoni L. Mentis G. Z. (2016). A stem cell model of the motor circuit uncouples motor neuron death from hyperexcitability induced by SMN deficiency. *Cell Rep.* 16 1416–1430. 10.1016/j.celrep.2016.06.087 27452470 PMC4972669

[B59] Singh R. N. Howell M. D. Ottesen E. W. Singh N. N. (2017). Diverse role of survival motor neuron protein. *Biochim. Biophys. Acta Gene. Regul. Mech.* 1860 299–315. 10.1016/j.bbagrm.2016.12.008 28095296 PMC5325804

[B60] Sleigh J. N. Dawes J. M. West S. J. Wei N. Spaulding E. L. Gomez-Martin A. (2017). Trk receptor signaling and sensory neuron fate are perturbed in human neuropathy caused by Gars mutations. *Proc. Natl. Acad. Sci. U.S.A.* 114 E3324–E3333. 10.1073/pnas.1614557114 28351971 PMC5402433

[B61] Soliven B. Maselli R. A. (1992). Single motor unit H-reflex in motor neuron disorders. *Muscle Nerve* 15 656–660. 10.1002/mus.880150604 1508230

[B62] Van Alstyne M. Simon C. M. Sardi S. P. Shihabuddin L. S. Mentis G. Z. Pellizzoni L. (2018). Dysregulation of Mdm2 and Mdm4 alternative splicing underlies motor neuron death in spinal muscular atrophy. *Genes Dev.* 32 1045–1059. 10.1101/gad.316059.118 30012555 PMC6075148

[B63] Wishart T. M. Mutsaers C. A. Riessland M. Reimer M. M. Hunter G. Hannam M. L. (2014). Dysregulation of ubiquitin homeostasis and beta-catenin signaling promote spinal muscular atrophy. *J. Clin. Invest.* 124 1821–1834. 10.1172/JCI71318 24590288 PMC3973095

[B64] Yonekawa T. Komaki H. Saito Y. Sugai K. Sasaki M. (2013). Peripheral nerve abnormalities in pediatric patients with spinal muscular atrophy. *Brain Dev.* 35 165–171. 10.1016/j.braindev.2012.03.009 22512990

[B65] Yuan P. Jiang L. (2015). Clinical characteristics of three subtypes of spinal muscular atrophy in children. *Brain Dev.* 37 537–541. 10.1016/j.braindev.2014.08.007 25199871

[B66] Zhang Y. Li N. Caron C. Matthias G. Hess D. Khochbin S. (2003). HDAC-6 interacts with and deacetylates tubulin and microtubules in vivo. *EMBO J.* 22 1168–1179. 10.1093/emboj/cdg115 12606581 PMC150348

[B67] Zhang Z. Pinto A. M. Wan L. Wang W. Berg M. G. Oliva I. (2013). Dysregulation of synaptogenesis genes antecedes motor neuron pathology in spinal muscular atrophy. *Proc. Natl. Acad. Sci. U.S.A.* 110 19348–19353. 10.1073/pnas.1319280110 24191055 PMC3845193

